# Disulfidptosis-related gene SLC3A2: a novel prognostic biomarker in nasopharyngeal carcinoma and head and neck squamous cell carcinoma

**DOI:** 10.3389/fonc.2025.1451034

**Published:** 2025-01-24

**Authors:** Xinyi Zhang, Yiqi Lin, Liang Shi, Aixia Zhai, Chao Wu, Qian-Ying Zhu

**Affiliations:** ^1^ Department of Laboratory Medicine, The Eighth Affiliated Hospital of Sun Yat-sen University, Shenzhen, China; ^2^ Department of Endocrinology, The Eighth Affiliated Hospital of Sun Yat-sen University, Shenzhen, China

**Keywords:** nasopharyngeal carcinoma, head and neck squamous cell carcinoma, SLC3A2, biomarker, disulfidptosis

## Abstract

**Introduction:**

Nasopharyngeal carcinoma (NPC), one of the most common malignancies of the head and neck, is characterised by a complex pathogenesis and an unfavourable prognosis. Recently, disulfidoptosis, a novel form of cell death, has been proposed. Several studies in recent years have extensively investigated the function of the disulfidoptosis-related SLC7A11 gene in cancer, but the role of its partner protein, SLC3A2, remains unknown unclear in NPC.

**Methods:**

GEO database analysis confirmed SLC3A2's prognostic impact on nasopharyngeal carcinoma. ROC, Kaplan-Meier analyses, and stage-specific expression studies showed a strong correlation with poor HNSC prognosis. GO and KEGG analyses pinpointed relevant signaling pathways. In vitro, SLC3A2's influence on cell proliferation, migration, and invasion was evaluated through CCK8, wound healing, colony formation, transwell assays, and cell cycle analysis.

**Results:**

In this study, we identified the high expression of SLC3A2 in NPC and head and neck squamous cell carcinoma (HNSC) and analyzed its potential mechanism and correlation with patient prognosis. Furthermore, a negative relationship was found between the expression level of SLC3A2 and the extent of immune cell infiltration and immune checkpoint. Differentially expressed genes (DEGs) between the high and low SLC3A2 expression groups were primarily involved in cytokine-cytokine receptor interaction and immune response. Finally, in vitro experiments demonstrated that SLC3A2 stimulates tumor cell proliferation and migration.

**Discussion:**

In conclusion, these results indicated a strong association between SLC3A2 and progression in both NPC and HNSC, suggesting it as a promising biomarker for predicting adverse prognosis in NPC and HNSC patients.

## Introduction

Head and neck squamous cell carcinomas (HNSC) originate from the mucosal epithelium in the oral cavity, pharynx and larynx (1). They are the most common malignancy of the head and neck (2). According to the 2020 Global Cancer Statistics report, HNSC is the eighth most common human cancer, with approximately 878,000 new cases and 444,000 deaths reported each year (3). Nasopharyngeal carcinoma (NPC) is a subtype of HNSC (4) that is more prevalent in southern China, Southeast Asia and North Africa (5). Radiotherapy and chemotherapy are the primary treatments for this cancer (6). However, NPC is usually diagnosed late, with local recurrence and distant metastasis (7), and the prognosis is poor (8). A recent investigation has redefined nasopharyngeal carcinoma as a multi-dimensional, space-time “ecological and evolutionary complex” within the pathological ecosystem (9). The authors elucidated that cancerous cells mimic invasive species, dynamically interacting with the tumor microenvironment (TME), immune responses, and a spectrum of other elements. They highlight that specific genes play a critical role in the ecological interactions within the TME. Therefore, the discovery of novel prognostic biomarkers and therapeutic targets for NPC and HNSC is particularly important (10).

Regulated necrosis, a gene-controlled cellular death process, emerges as a crucial contributor to tissue growth, homeostasis and pathological activities (11). Existing research has already demonstrated the involvement of cell death in cancer occurrence, development, metastasis, and prognosis (12). Lately, a new form of cell death termed disulfidptosis has been proposed (13). Under conditions of glucose starvation, cells that overexpress SLC7A11 experience severe NADPH depletion and abnormal accumulation of cystine and other disulfides, leading to disulfide stress. This stress is highly toxic to cells and can affect the survival and proliferation of cancer cells (14). After genome-wide CRISPR/Cas9 screening, the chaperone protein SLC3A2 ranked second on the list of genes required for disulfidptosis (i.e. knocking out the gene inhibits disulfidptosis) (15). In recent years, there has been significant research progress on the function of SLC7A11 in cancer (16). However, very few studies have examined the role of its partner, SLC3A2, particularly in NPC and HNSC patients.

To explore possible pathogenic mechanisms, we initially utilized two data entities, the Gene Expression Omnibus (GEO) database and the Cancer Genome Atlas (TCGA) database. Through these databases, we evaluated the expression levels of 10 genes related to disulfidptosis in NPC. Our findings revealed that SLC3A2 showed elevated expression in NPC tissues, which correlated with an adverse prognosis in NPC and HNSC patients. Additionally, a negative association was observed between the expression of SLA3A2 and immune cell infiltration as well as the checkpoint. Furthermore, the differential expression genes (DEGs) in the high- and low-expression groups of SLC3A2 were significantly concentrated in cytokine-cytokine receptor interaction and immune response, as indicated by functional enrichment analysis. Finally, our study confirmed the oncogenic role of SLC3A2 in the proliferation and migration of tumor cells *in vitro*. In short, our investigation showed that SLC3A2 could be a novel prognostic biomarker for patients with NPC and HNSC, emphasizing its significant role in the progression of these cancers.

## Materials and methods

### Data collection and processing

We gathered the integrated transcriptome expression matrices and clinical information for NPC and HNSC from the Gene Expression Omnibus (GEO) and The Cancer Genome Atlas (TCGA) databases. Clinicopathological characteristics of SLC3A2 in HNSC samples were validated with TCGA datasets. For additional analysis, we utilized the GSE102349 dataset (update date: May 15, 2019), which included mRNA expression data and clinical information from 113 NPC patients, and 88 cases with progression-free survival (PFS).

### Detection of disulfidptosis-related regulators expression variations in NPC

For differential expression analysis, 10 disulfidptosis-related genes were selected. We employed the keyword “nasopharyngeal carcinoma” to search the GEO database for suitable datasets. Following that, we manually reviewed the datasets and selected those that included comprehensive RNA expression data with large sequencing sample sizes, including GSE12452 (encompassing 10 healthy nasopharyngeal samples and 31 NPC samples, update date: Mar 25, 2019) and GSE53819 (encompassing 18 healthy nasopharyngeal samples and 18 NPC samples, update date: Aug 01, 2019). We assessed the differential expression of these genes in NPC by conducting the Wilcoxon test on the two datasets.

### Survival analysis

Utilizing R software package, Survival and survminer, we examined the survival rate. Among the 113 patients in GSE102349, a total of 88 patients, who provided detailed progression-free survival information, were included in the survival analysis. We used the Kaplan-Meier method to produce the survival curves and the log-rank test to determine their significance.

### Creation and verification of nomogram

We constructed a nomogram that combined T stage, N stage and SLC3A2 expression to evaluate 3- and 5-year prognostic characteristics. Every patient’s clinical data has a particular grade, and the cumulative grade was an aggregation of individual indices within the nomogram scoring mechanism. Calibration curves were used to predict survival probability.

### Investigation of immune infiltration of HNSC

The ESTIMATE algorithm was used to calculate the immune and stromal scores, which aided in identifying gene signature abundance from stromal and immune cells. We examined the enrichment score of 24 immune cells and 15 immunosuppressive checkpoints using the R package GSVA.

### Generation of DEGs in SLC3A2-high and low expression groups

Based on survival analysis results, 88 patients from GSE102349 were classified as having high or low SLC3A2 expression levels. The limma package was utilized for pinpointing the DEGs between these groups. Significant DEGs were determined by a |logFC| exceeding 0.8 and a false discovery rate (FDR) under 0.05.

### Single-cell database analysis

TISCH collected data from Gene Expression Omnibus (GEO) and ArrayExpress to formulate its scRNA-seq atlas. TISCH includes 79 databases and 2045746 cells from both tumor patients and healthy donors. The datasets were uniformly processed to enable clarifying components of the TME at both single-cell and annotated cluster levels. In this work, we investigated SLC3A2 expression in TME-associated cells using two nasopharyngeal carcinoma datasets (GSE150430 and GSE162025) from the TISCH database.

### Validation of the protein expression levels

To further verify the protein expression levels of SLC3A2 in HNSC tissues, immunohistochemistry (IHC) data was downloaded from the Human Protein Atlas (HPA, http://www.proteinatlas.org). The HPA could provide IHC results of multiple proteins based on proteomics in both cancer tissues and normal tissues.

### Investigation of Functional Enrichment

Functional enrichment analysis leveraged gene ontology (GO) and Kyoto Encyclopedia of Genes and Genomes (KEGG) to delve deeper into possible gene functions and enrichment pathways related to SLC3A2. The GO analysis encompasses three categories: biological process (BP), cellular component (CC) and molecular function (MF).

### Reagents and cell culture

The human NPC cell lines CNE1 and CNE2 were cultured in RPMI-1640 (Gibco, Thermo Fisher Scientific) supplemented with 5% fetal bovine serum (FBS, HUAYUN). The cells were incubated in a constant temperature oven at 37°C with 5% CO2. All NPC cell lines, which had been authenticated, were kindly provided by Dr. Mu-Sheng Zeng (Sun Yat-sen University Cancer Centre).

### siRNA transfection

The small interfering RNA (siRNA) targeting SLC3A2 (si-SLC3A2, 5’‐ GGATGAGATTGGCCTGGAT‐3’) was produced by RiboBio (Guangzhou, China). The transfection process for the siRNA was executed using Lipo6000 (Beyotime), in accordance with the supplied guidelines. In short, predetermined cells were seeded in a 6-well plate for 12 hours before being transfected with 20nM siRNA mixed with 5μL Lipo6000. Cells were collected 48 hours post-transfection for subsequent experimentation. The knockdown efficiency of SLC3A2 was verified by western blotting.

### Western blot

Protein samples were obtained from lysis of cells extracted by sample buffer (Beyotime). The proteins were separated by molecular weight using sodium dodecyl sulfate-polyacrylamide gel electrophoresis (SDS-PAGE). The isolated proteins were transferred from the gel to a polyvinylidene fluoride (PVDF) membrane (Merck Millipore) and blocked with 5% skim milk for one hour at room temperature. The blots were cut and then incubated with the primary antibodies of anti-SLC3A2 (CST-13180S, 1:1,000 dilution), rabbit anti-GAPDH (Servicebio-GB15004, 1:3,000 dilution) respectively at 4°C overnight. After three times of washing with TBST, the membranes were incubated with HRP-conjugated goat anti-rabbit IgG (Beyotime-A0208, 1:3,000 dilution) for an hour at room temperature. Following three additional TBST rinses, the strips were exposed to a chemical luminescent agent (GBCBIO) for imaging detection. The protein bands were analyzed using Image Lab (version 3.0) to quantify the gene knockdown efficiency at the translational level. All blots with clear membrane edges were provided in the **Supplementary Figure 1**.

### Cell proliferation and clone formation assays

Cell proliferation was evaluated using the Cell Counting Kit-8 (CCK8, Biosharp) and colony formation tests. Two types of NPC cell populations, CNE1 and CNE2, underwent siRNA transfection. After 48 hours, 1,000 transfected cells were seeded into the 96-well plate per well. For the following four days, the old medium was substituted with a new one containing 10% CCK8 solution. The plates were then incubated for two hours at 37°C before the OD value at 450 nm was recorded.

In the clonogenic assay, transfected cells were seeded in a 6-well plate (1000/well) and cultured for two weeks with medium changes every five days. The medium was then evacuated, and the cells were washed with PBS twice and fixed in 4% paraformaldehyde for 30 minutes. Finally, cells were dyed with 0.1% crystal violet (Beyotime) for 10 minutes before the colonies were counted and photographed.

### Wound healing assay

Transfected NPC cells were placed on a 6-well plate and incubated at 37°C in 5% FBS-1640 medium until complete fusion without overgrowth. Three horizontal scratches were made about 1/4 of the way across the plate using a marker. An 1mL pipette tip was used to make a vertical scratch in the center of each well with constant pressure. The cells were rinsed once with PBS and then the medium was changed to serum-free. Phase contrast imaging of the wound was performed using an inverted microscope. After 24 hours, fresh serum-free medium replaced the medium on the cells, and the images were taken again. The crossing points of scratches were used to ensure that the images were taken at the same location. Image J (fiji) was used to measure wounds.

### Transwell migration analysis

For the migration assay, 8 × 10^5^ transfected NPC cells were distributed in 200µl of serum-free medium in the upper chamber. The lower chamber received 500µl of complete medium containing 10% FBS. After 24 hours, non-migratory cells in the upper compartment were eliminated, and the migrated cells beneath the surface were fixed with 4% paraformaldehyde for 30 minutes and then stained with 0.1% crystal violet for 10 minutes. After rinsing off any excess crystal violet, we randomly selected five areas and counted the cells using a Leica microscope.

### Cell cycle analysis

Flow cytometry technique used to analyze the progression of the cell cycle. The CNE cells were incubated for 72 hours at 37°C. After incubation, the cells were washed with ice-cold PBS, trypsinized, collected, and pelleted by centrifugation at 3000 rpm for 3 minutes. The pellets were then washed twice with ice-cold PBS and fixed in 70% ice-cold ethanol overnight. Subsequently, the cell suspension was centrifuged, and the cells were washed in PBS and re-suspended in 500 µL of PBS containing 50 µg/mL propidium iodide (PI, KeyGEN, KGA9101-50) and 0.1 mg/mL RNase A, and incubated in the dark for 30 minutes at room temperature. Flow cytometry analyses were performed using a BD LSR Fortessa Cell Analyzer. In each sample, approximately 10,000 cells were analyzed. The data obtained were analyzed using ModFit LT 5.0 software.

### Statistical analysis

R language software (version 4.2.3) was used to analyze the data. T-tests were performed for data with normal distribution, while Spearman’s test was used for relevant analyses. GraphPad Prism software (version 9.5) was used to generate graphs. A P value of less than 0.05 indicates a statistically significant result (*P < 0.05, **P < 0.01, ***P < 0.001 and ****P < 0.0001), while ns indicates no significance.

## Results

### Expression profile of disulfidptosis-related genes and its correlation with prognosis in NPC

**Figure 1:**
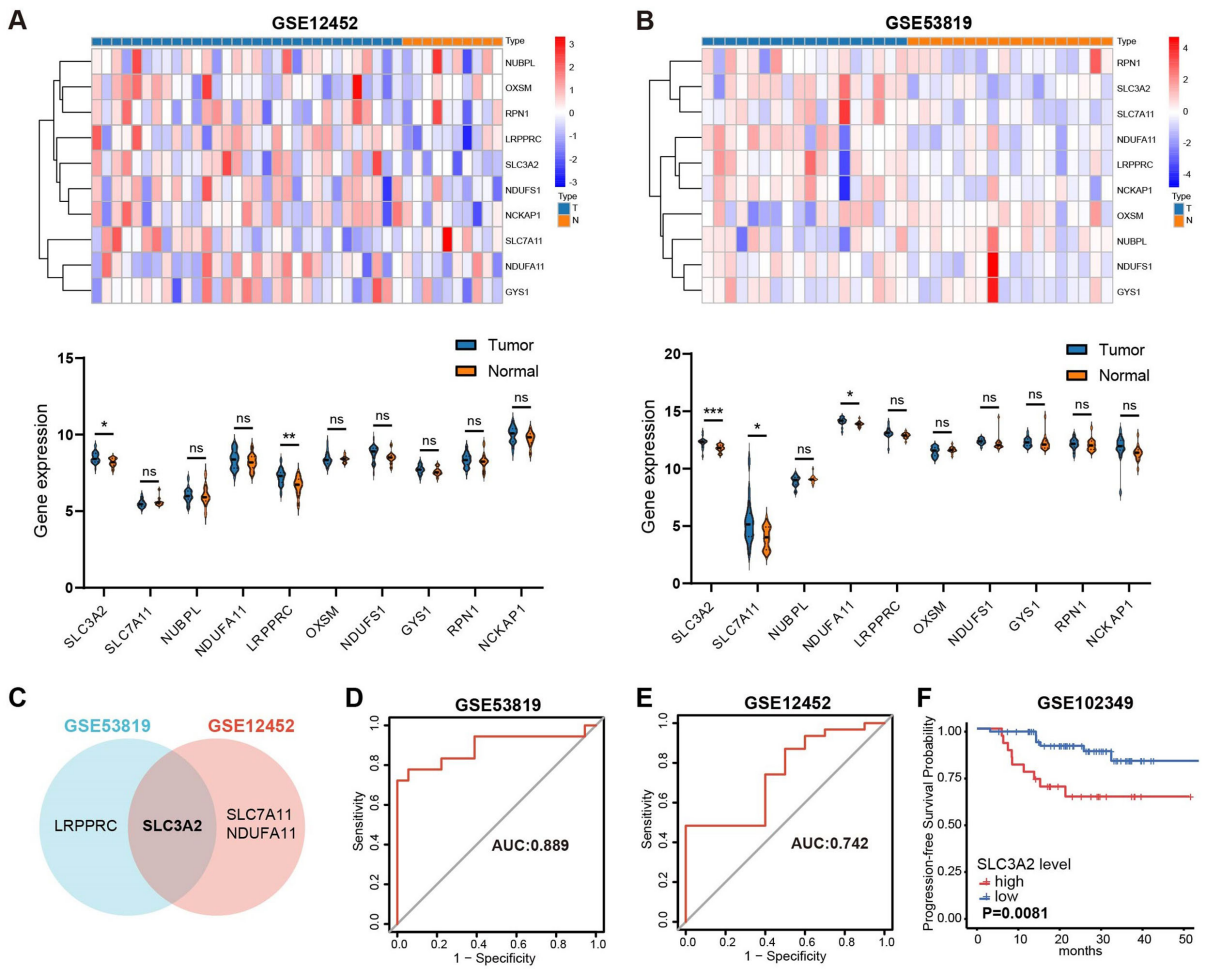
Overview of the expression patterns of disulfideptosis-associated genes and their relationship with NPC patient survival. The expression levels of disulfidptosis-related genes in GSE12452 **(A)** and GSE53819 **(B)**. **(C)** The common differentially expressed disulfidptosis-related genes in GSE53819 and GSE12452. ROC curve analysis for SLC3A2 diagnosis in GSE53819 **(D)** and GSE12452 **(E)**. **(F)** Kaplan–Meier curves of PFS for high- and low-SLC3A2 expression groups in GSE102349. *P value< 0.05, **P value< 0.01, and ***P value< 0.001, ns, no significant.

A recent study uncovered 10 disulfidptosis-related genes, namely SLC7A11, SLC3A2, GYS1, NDUFA11, LRPPRC, NUBPL, NCKAP1, OXSM, RPN1 and NDUFS1 (17). Two GEO datasets, GSE53819 and GSE12452, were used to investigate whether disulfidptosis-related genes are aberrantly expressed in NPC. Revealed in **Figures 1A–C**, LRPPRC and SLC3A2 showed elevated expression in NPC in comparison to normal tissue in GSE53819; similarly, NDUFA11, SLC7A11 and SLC3A2 displayed increased expression in GSE12452. Thus, SLC3A2 was selected for further investigation. The receiver operating characteristics (ROC) curves of SLC3A2 for distinguishing NPC from the control group showed the AUC of 0.889 in GSE53819 (**Figure 1D**) and 0.742 in GSE12452 (**Figure 1E**). As shown in **Figure 1F**, high SLC3A2 levels indicated poor prognosis for NPC patients in GSE102349. Taken together, the disulfidptosis-related genes were misexpressed in NPC, and SLC3A2 was expected as a prognostic predictor for NPC patients.

### Relationship between SLC3A2 and Clinicopathological Characteristics in HNSC

**Figure 2:**
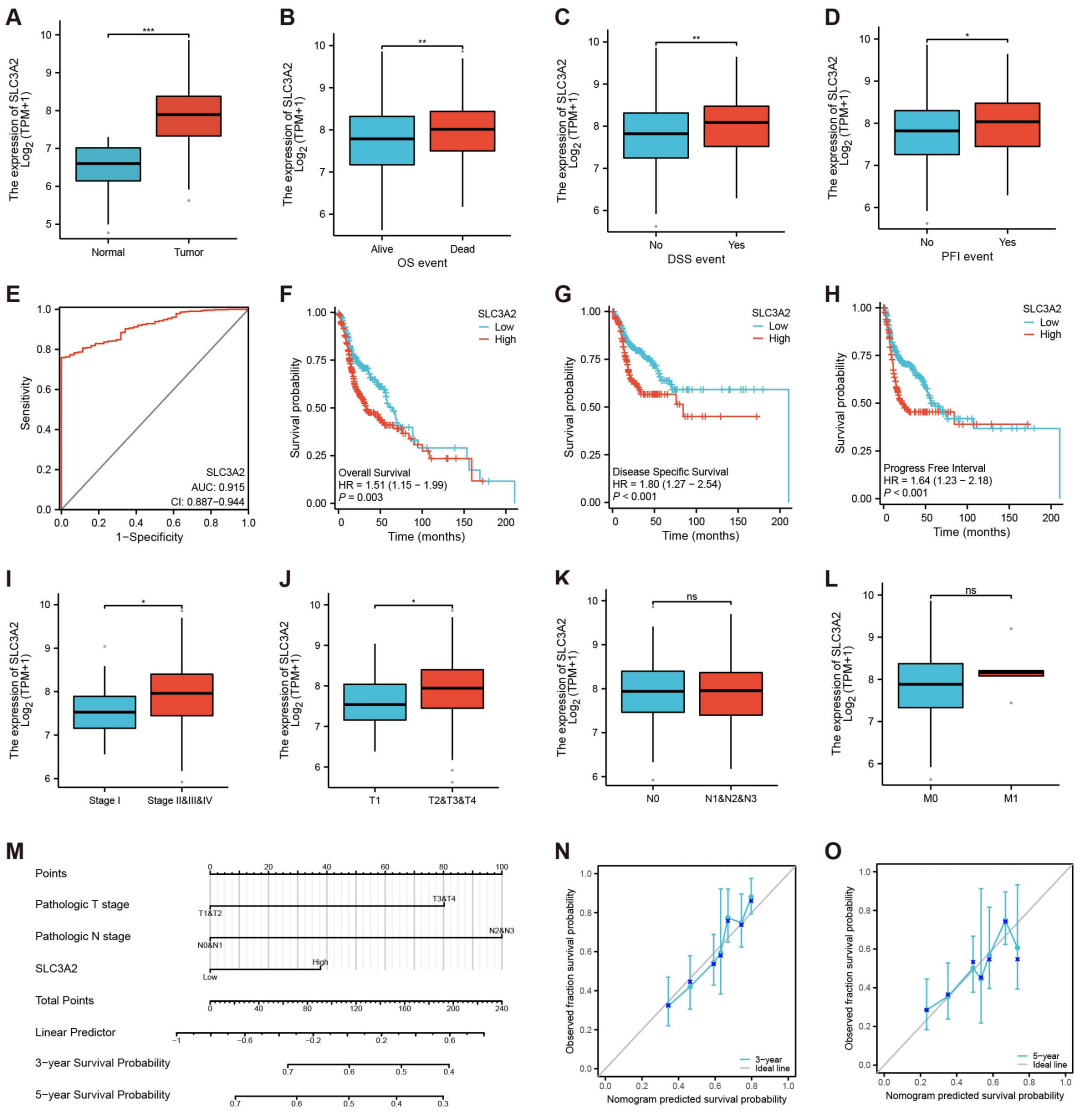
Identification of the clinical pathology and prognostic characteristics concerning SLC3A2. **(A–D)** Different expressions of SLC3A2 in different clinical stages (**A**: Tissue, **B**: OS, **C**: DSS, **D**: PFI, Yes: the endpoint event has occurred, No: the endpoint event hasn’t occurred). **(E)** ROC curve of SLC3A2 in HNSC. **(F–H)** Survival analysis of SLC3A2 in OS, DSS and PFI. **(I–L)** Differential expression of SLC3A2 in diverse pathological phases (**I**: Stage, **J**: T, **K**: N, **L**: M). **(M)** Nomogram model construction utilizing independent prognostic factors to predict the 3- and 5-year survival probabilities for patients with HNSC. **(N–O)** The calibration curves for predicting the survival probabilities for patients with HNSC (N: 3-years, O: 5-year). *P value< 0.05, **P value< 0.01, and ***P value< 0.001, ns, no significant.

In the context of HNSC, we found that SLC3A2 was markedly overexpressed in tumor tissues compared to normal controls (**Figure 2A**). Besides, HNSC patients with tumor progression showed higher SLC3A2 expression (**Figures 2B–D**). In addition, ROC curve analysis showed that it displayed a diagnostic potential with an AUC of 0.915 (**Figure 2E**). We then investigated the correlation between SLC3A2 and patient outcome. Survival analysis revealed that high expression of SLC3A2 was linked to poorer overall survival (OS), disease-specific survival (DSS) and progression-free interval (PFI) in HNSC patients (**Figures 2F–H**). Further investigation of SLC3A2 expression across varying clinicopathological stages showed a positive link to the pathological T stage and N stage in HNSC (**Figures 2I–L**). We then constructed a nomogram based on defined prognostic factors consisting of pathological T stage and N stage and the SLC3A2 expression for the prognostic prediction of patients with HNSC. The calibration curves indicated a robust consistency between the predictions of the nomogram and the recorded survival rates in both 3-year and 5-year models (**Figures 2M–O**). These findings outlined the importance of SLC3A2 as a prognostic marker for HNSC.

### Elucidation of Immunological Features of the SLC3A2

**Figure 3:**
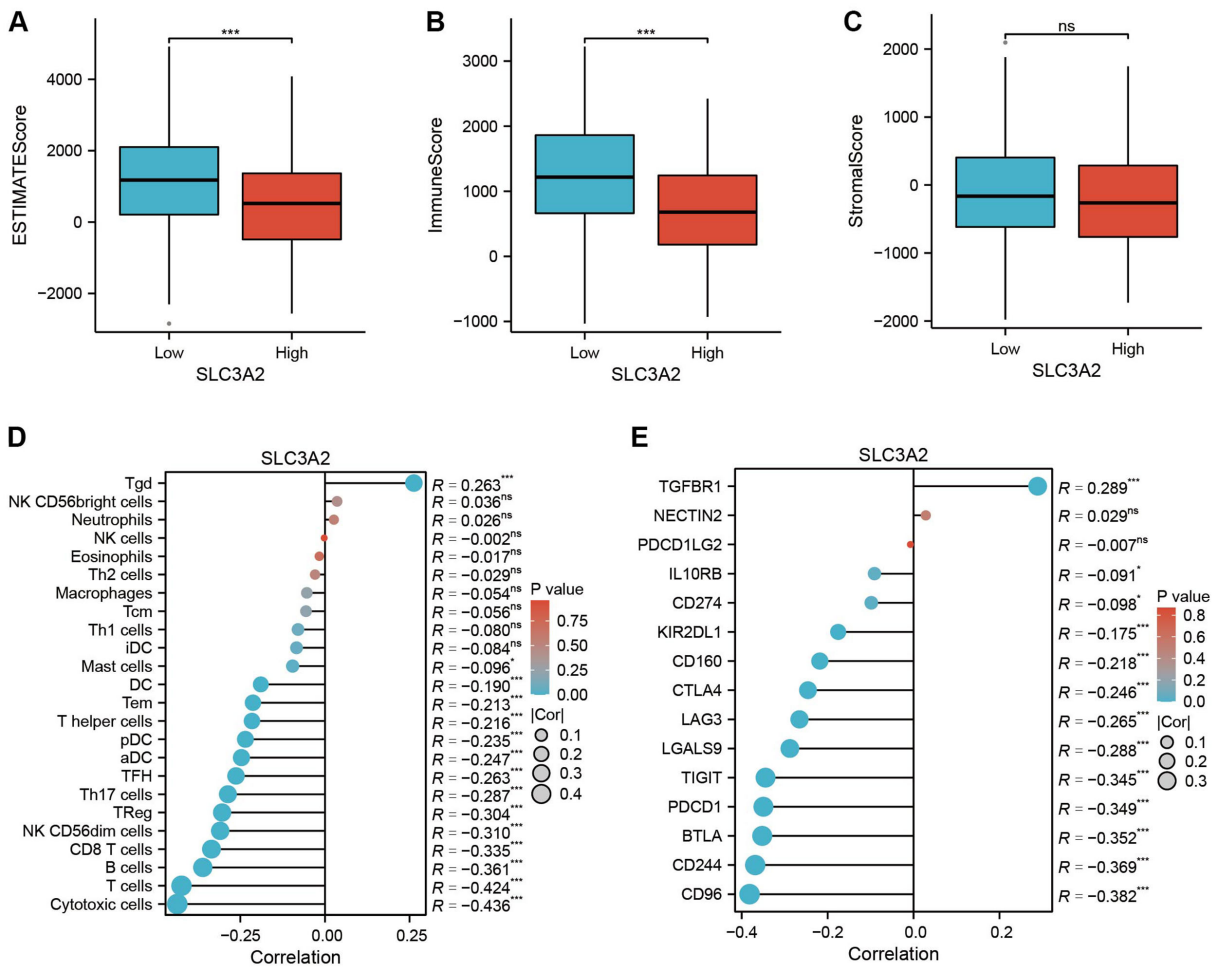
**(A–C)** Differential expression of tumor microenvironment scores (ESTIMATEScore, ImmuneScore and StromalScore) between high and low SLC3A2 groups. **(D)** Association analysis between SLC3A2 and immune infiltrating cells. **(E)** Correlation analysis between SLC3A2 and immunosuppressive checkpoints. *P value < 0.05, ***P value < 0.001, ns, no significant.

To examine the link between SLC3A2 expression patterns and the immune microenvironment, HNSC patients were divided into high and low SLC3A2 expression groups based on the median SLC3A2 expression value. Our study unveiled a connection between SLC3A2 expression and immune microenvironmental characteristics, as well as a negative connection with immuneScore and estimatScore (**Figures 3A–C**). Moreover, SLC3A2 expression was found to inversely correlated with the abundance of suppressive immune cells, including cytotoxic cells, T cells, B cells and CD8^+^ T cells (**Figure 3D**). In addition, an inverse correlation was found between SLC3A2 expression and the abundance of immunosuppressive checkpoints such as CD96, CD244, BTLA and PDCD1 (**Figure 3E**). These findings suggested that higher levels of SLC3A2 expression are associated with an immunosuppressive microenvironment in HNSC, which is characterized by increased levels of immunosuppressive cells and altered expression of immunosuppressive checkpoints.

### SLC3A2 potentially influences the infiltration of immune cells in the tumor microenvironment

**Figure 4:**
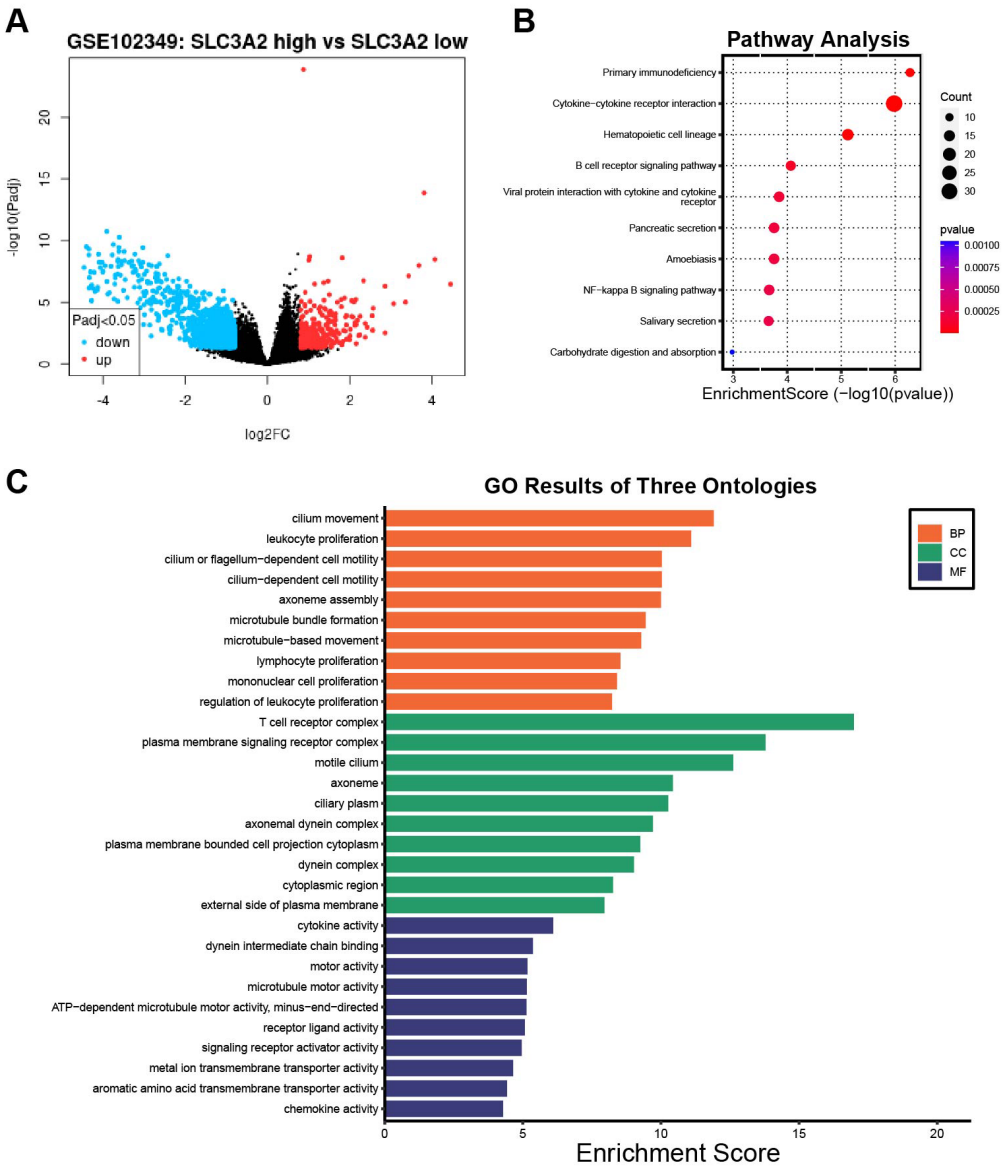
The differentially expression analysis of samples with SLC3A2 high and low expression. **(A)** The volcano plot of DEGs between the SLC3A2 high expression and low groups in GSE102349. KEGG pathway analysis **(B)** and GO functional annotation analysis **(C)** of DEGs in connection with SLC3A2 high and low expression groups.

To investigate the oncogenic process of SLC3A2 in NPC, we analyzed a cohort of 88 patients from GSE102349 and divided them into low SLC3A2 expression (n = 62) and high SLC3A2 expression (n = 26) groups. Comparisons between high and low SLC3A2 expression samples were performed using transcriptome analysis. **Figure 4A** revealed that 325 genes were enhanced and 1238 genes were reduced (|logFC| ≥0.8 and P <0.05). The top tier of upregulated genes included SLC3A2, LRP2, EEF1AKMT4-ECE2, ASTL and CPA2, while the most notably downregulated genes were VWA3A, KIAA2012, VWA3B, CDHR4 and PIFO. The KEGG pathway analysis was performed on the differential expression genes (DEGs), revealing that the DEGs between the high and low SLC3A2 groups were primarily enriched in primary immunodeficiency, cytokine-cytokine receptor interaction, and B cell receptor signaling pathways (**Figure 4B**). Additionally, the GO enrichment analysis revealed that these DEGs massively converged on immune response pathways, such as cytokine activation, lymphocyte and mononuclear cell proliferation (**Figure 4C**). These findings suggested that SLC3A2 levels may affect the infiltration of immune cells within the TME in NPC.

### SLC3A2 promotes NPC cell proliferation and migration *in vitro*

**Figure 5:**
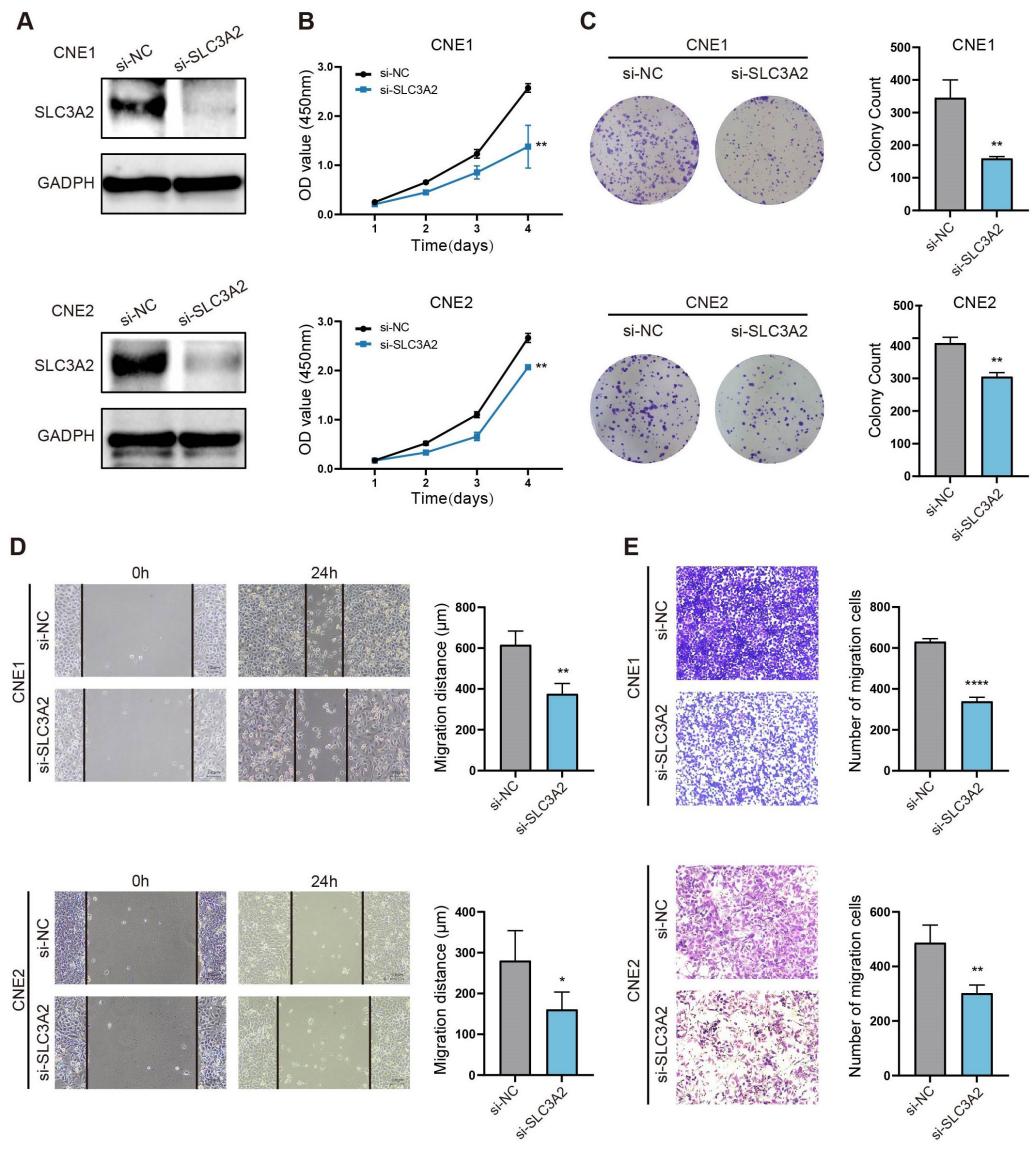
SLC3A2 enhances the proliferation and migration of NPC cells. **(A)** Western blot analysis indicates the efficiency of SLC3A2 knockdown in CNE1 and CNE2 cells. GAPDH as an internal reference. The effects of knockdown of SLC3A2 on the proliferation of NPC cells were evaluated by **(B)** CCK8 and **(C)** clone formation assay. The effect of SLC3A2 knockdown on NPC cell migration was observed by wound healing assays **(D)** and transwell assays **(E)**. Mean ± SEM. Student’s t-test. *P value< 0.05, **P value< 0.01 and ****P value< 0.0001.

The likely biological role of SLC3A2 in NPC progression was explored by administering SLC3A2 knockdown to two NPC cell lines (CNE1 and CNE2) with siRNA. SLC3A2 expression was notably diminished in these two NPC cell lines following transfection (**Figure 5A**). The knockdown efficiency is greater than 70% after the strip gray detection (**Supplementary Figure 2**). Moreover, CCK-8 tests displayed a significant hindrance of NPC cell propagation after SLC3A2 knockdown (**Figure 5B**). We analyzed the distribution of cell cycle phases by measuring the DNA content of SLC3A2 knockdown and control cells using flow cytometry (**Supplementary Figure S5**). Compared to the control group, cells with SLC3A2 knockdown exhibited an increase in the G2/M phase and a decrease in the G1 phase. In conjunction with the results of the cell proliferation assays, we hypothesize that SLC3A2 knockdown may arrest cells in the G2/M phase, thereby leading to reduced proliferation. This is consistent with the findings reported in previous literature (18). To assess the persistent influence of SLC3A2 on NPC cells reproduction, colony formation assessments were conducted, and a reduced number of colonies was found (**Figure 5C**). Given the strong association between SLC3A2 expression and NPC pathological stages, it is presumed that SLC3A2 significantly impacts NPC cell migration ability. The wound healing assay demonstrated that cell migration was blocked by knocking down of SLC3A2 (**Figure 5D**). The transwell assay indicated that suppressing SLC3A2 in NPC cells decreased the number of cells traversing the chamber (**Figure 5E**). Summarily, these findings denoted that SLC3A2 could enhance the proliferation and migration of NPC cells *in vitro*.

## Discussion

NPC has unique biological, epidemiological and etiological characteristics. Due to its high aggressiveness, poor prognosis, and early-stage subtle symptoms, approximately 70% of NPC patients being diagnosed at an advanced stage (19, 20). Although radiotherapy- and chemotherapy-based therapies have made significant advances in combating non-metastatic NPC (21, 22), distal metastasis is still the main reason for death amongst these patients (23). Consequently, the search for reliable tumor markers is crucial for the early detection and molecular targeted therapy of NPC. Disulfidptosis is a novel form of cell death that is distinct from the known cell death forms such as copper death, iron death, pyroptosis, apoptosis and necrosis (24–27). The condition is characterized by an abnormal and excessive buildup of disulfide in cells. This leads to an increase in the content of disulfide bonds in the actin skeleton, causing actin filament contraction, destruction of the cytoskeletal structure, and ultimately cell death (17). Disulfide bonds are important regulators that can alter crucial cell activities, including the survival and metastasis of tumor cells (28).

It has been confirmed that disulfide death plays a role in hepatocellular carcinoma (29) and renal cell carcinoma (30). Previous studies have reported that the disulfidptosis-related gene SLC7A11 regulates expression in most cancers and mediates multiple regulatory mechanisms (31, 32). However, the expression level and biological function of SLC3A2, the chaperone protein-coding gene of SLC7A11 (33), in head and neck malignancies, particularly NPC, have not been extensively studied in the existing literature.

Using datasets GSE53819 and GSE12452, our recent study revealed the expression patterns of genes associated to disulfidptosis in NPC and non-pathological tissues. The intersection of the two datasets revealed that SLC3A2 was upregulated in NPC tissues compared to normal tissues. Additionally, ROC curves for SLC3A2 in the two datasets revealed accuracy values of 0.889 and 0.742. Increased expression of SLC3A2 has been linked to a bad prognosis for NPC patients in the GSE102349 dataset, according to a subsequent survival analysis. Therefore, we selected SLC3A2 as the target gene for our following research.

Since NPC is a subtype of HNSC (34), we selected HNSC-related datasets from the TCGA for further analysis. In this study, we explored the potential mechanism of SLC3A2 and its impact on patient outcomes in the context of HNSC. Through ROC curve analysis, Kaplan-Meier analysis, and expression profile studies in different clinicopathological stages, the results implied that SLC3A2 is significantly related to poor prognosis in HNSC. In addition, a nomogram was constructed using SLC3A2 expression levels in HNSC to forecast patient survival at 3- and 5-year intervals. The calibration maps indicated high predictive value. Therefore, it was suggested that SLC3A2 is associated with disease progression and could be a potential prognostic biomarker for HNSC.

Historically, tumor progression was thought to be caused only by genetic and epigenetic changes in tumor cells (35). However, recent researches have shown that the TME composed of various cell populations, including inflammatory cells, glial cells, cancer-associated fibroblasts, and various infiltrating immune cells, plays an important role in tumor progression (36, 37). The implications of these factors are significant for the development of cancer, including immune detection and destruction, evasion of apoptosis, and activation of invasion and metastasis (38–40).

Therefore, we evaluated the proportion of stromalScore, immuneScore and ESTIMATEScore in groups with high and low expressions of SLC3A2. We found a significant increase in the immuneScore and ESTIMATEScore amongst the SLC3A2-low group, suggesting a greater presence of immune aspects in HNSC patients within this category. Furthermore, immunoinfiltration is strongly linked to the response to immunotherapy (41, 42). To enhance the effectiveness of existing immunotherapies and formulate new strategies, predicting immunotherapy response based on the infiltrating properties of the tumor microenvironment is crucial (43, 44). Thus, in the two HNSC groups, we examined the extent of penetration of 24 immune-associated cell types. We discerned that SLC3A2 expression has a negative relation with the abundance of immune inhibitory cells, inclusive of T cells, B cells, CD8^+^ T cells and regulatory T cells. Immune checkpoint medicines have seen notable progress in recent years, with regards to innovative treatment developments (45–47). Therefore, we measured the expression levels of frequently observed immune checkpoint genes and discovered that the majority of them correlated negatively with the expression of SLC3A2.

NPC is an aggressive epithelial malignancy that is marked by EBV infection and severe lymphocyte infiltration (37). This suggests that TME significantly contributes to its development (48). Cancer cells benefit from a metabolic advantage, excelling in nutrient uptake and contributing to a hostile TME. This environment poses significant challenges for cytotoxic immune cells, impeding their ability to adapt, infiltrate tumors, survive, and ultimately eradicate cancer cells. Previous studies have found that the expression of SLC3A2 is linked to the activation of important metabolic pathways, such as mTORC1 (a metabolic regulator that promotes cell metabolism) and c-Myc (which promotes cell growth, proliferation, and survival) (49), so we speculate that SLC3A2 may affect the progression and prognosis of NPC and HNSC by influencing immune cell infiltration in TME. To validate our conjecture, we segmented NPC patients into SLC3A2 low and high expression groups from GSE102349. The GO and KEGG enrichment analyses demonstrated that the DEGs in the two groups were mainly focused on immune responses, such as cytokine-cytokine receptor interaction, immune cell receptor signaling pathways, leukocyte activation, and lymphocyte and monocyte proliferation. Taken together, these results suggest that SLC3A2 may enhance the metabolism of tumor cells, thereby affecting the function of cytokines and the infiltration of immune cells into nasopharyngeal carcinoma tissues, and ultimately promote the progression of tumor. TME-based immunotherapy has also drawn attention as a potential treatment for NPC (50). Contemporary anti-tumor immunotherapies, including CAR-NK/T cells that are designed to enhance tumor recognition, often face metabolic disadvantages in the nutrient-scarce and hostile TME (49). Our observations indicate that targeting SLC3A2 could potentially enhance the efficacy of immunotherapies by modifying the cytokine milieu and facilitating improved infiltration of immune cells into tumors. Taken together, these findings represented a promising future direction for the combination of immunotherapy and targeted SLC3A2 therapy in HNSC and NPC patients.

Meanwhile, it was first observed that SLC3A2 can enhance the proliferation and migration of NPC cells *in vitro*, demonstrating its substantial role in increasing the carcinogenicity of NPC cells. This finding was consistent with previous research on other tumors (49, 51, 52). Previously, SLC3A2 had been reported to modulate integrin, PI3k/Akt and MEK/ERK signaling pathways. By binding to intracellular domains of β integrin, SLC3A2 modulates integrin signaling resulting in alterations of cell proliferation, adhesion and migration (51). Evidence suggests that SLC3A2, as an amino acid transporter, participates in sensing amino acid levels and thereby activates mTORC1, a key metabolic regulator that promotes cell metabolism and induces the expression of c-Myc, a transcription factor essential for cell growth and proliferation (49).

Given the significant role of the TME in cancer progression and its impact on prognosis, we combined various datasets to investigate the distribution of SLC3A2 expression within the TME. Initially, we examined SLC3A2 expression in TME-associated cells using two nasopharyngeal carcinoma datasets (GSE150430 and GSE162025) from the TISCH database. Our findings revealed that SLC3A2 is expressed in both malignant tumor cells and immune cells (**Supplementary Figure 3**). Secondly, we analyzed immunohistochemistry data from the Human Protein Atlas database, which indicated that SLC3A2 is predominantly localized on tumor cell membranes (**Supplementary Figure 4**). Therefore, we proposed that SLC3A2 could impact the progression and prognosis of NPC and HNSC by both influencing immune cell infiltration and directly affecting tumor cells.

However, our research has some restrictions. First, we investigated disulfidptosis-related genes relying on public databases such as GEO and TCGA, primarily using Western case data, lacking forward-looking representative data. Furthermore, we have not investigated whether disulfiptosis occurs in NPC cells, nor have been verified with clinical samples, and the specific mechanism of action of SLC3A2 has not been explored deeply enough. Our future research plan includes conducting immunostaining experiments to verify disulfiptosis in nasopharyngeal carcinoma. Additionally, we intend to recruit eligible patients in our hospital to further substantiate the reliability and accuracy of the results obtained in this study. We expect to supplement clinical validation experiments and establish animal models to confirm the authenticity and reliability of the study results in humans, clarify the effect of SLC3A2 on tumor progression *in vivo*, and further explore the specific mechanism of action of SLC3A2 in tumor progression.

In summary, we discovered that the disulfidptosis-related gene SLC3A2 exhibits high expression levels in NPC and HNSC tissues, and its presence is closely linked to tumor stage, which could predict poor prognosis in these patients. In addition, SLC3A2 was found to have an inverse relationship with the proliferation and infiltration of multiple immune cells in TME. Moreover, *in vitro* experiments demonstrated that SLC3A2 significantly enhances the malignant phenotype of NPC cells. To sum up, our study provided fresh perspectives for understanding the molecular mechanisms of NPC and HNSC pathogenesis and discovered a novel prognostic biomarker for these cancers.

## Data Availability

The original contributions presented in the study are included in the article/**Supplementary Material**. Further inquiries can be directed to the corresponding authors.
