# Supplementary material for: Disulfidptosis-related gene SLC3A2: a novel prognostic biomarker in nasopharyngeal carcinoma and head and neck squamous cell carcinoma
Source: Front Oncol. 2025 Jan 24;15:1451034. doi: 10.3389/fonc.2025.1451034 (PMC11802814; doi:10.3389/fonc.2025.1451034)
Supplement: Supplementary file 1 [file DataSheet1.docx]

Supplementary Material

1. **Supplementary Figures**


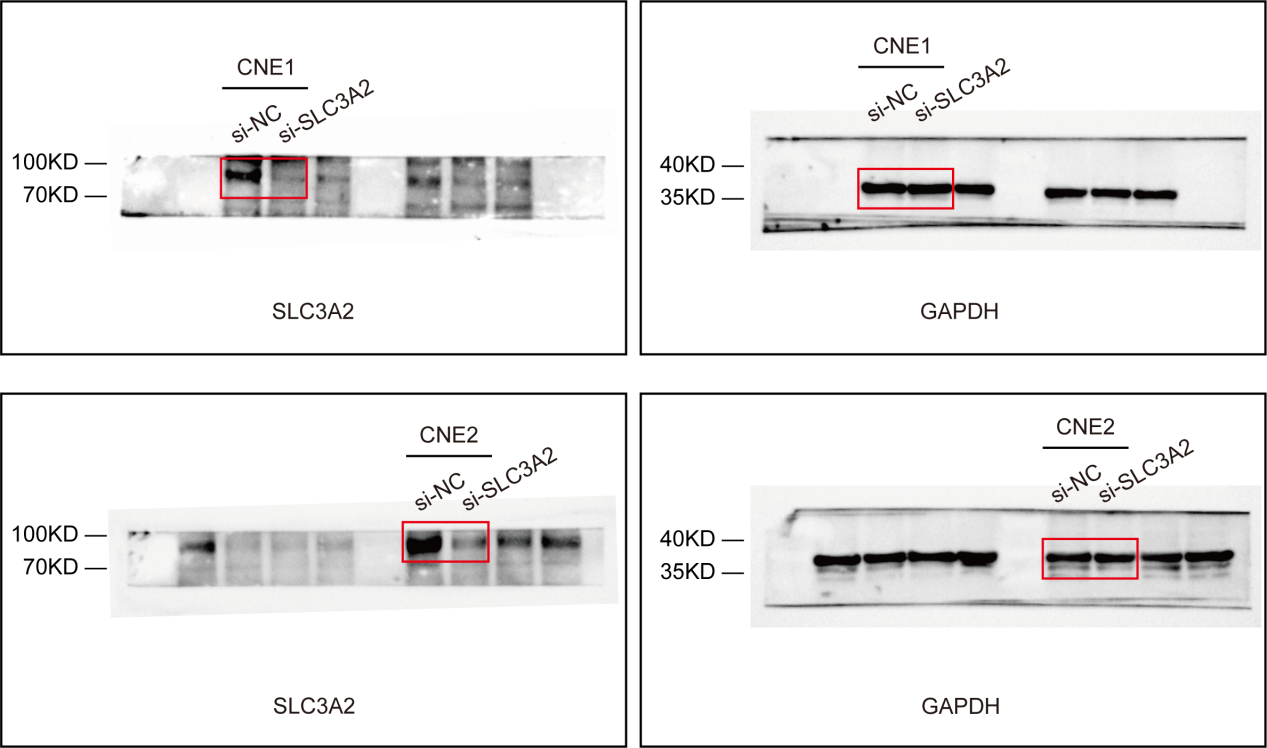


**Supplementary Figure 1**. Western blot analysis indicates the efficiency of SLC3A2 knockdown in CNE1 and CNE2 cells. GAPDH as an internal reference. Complete images from blots used throughout the manuscript with the corresponding molecular weight markers are shown. Cropped areas in every blot are marked and the antibody used in each case is named. Related to **Figure 5A**.


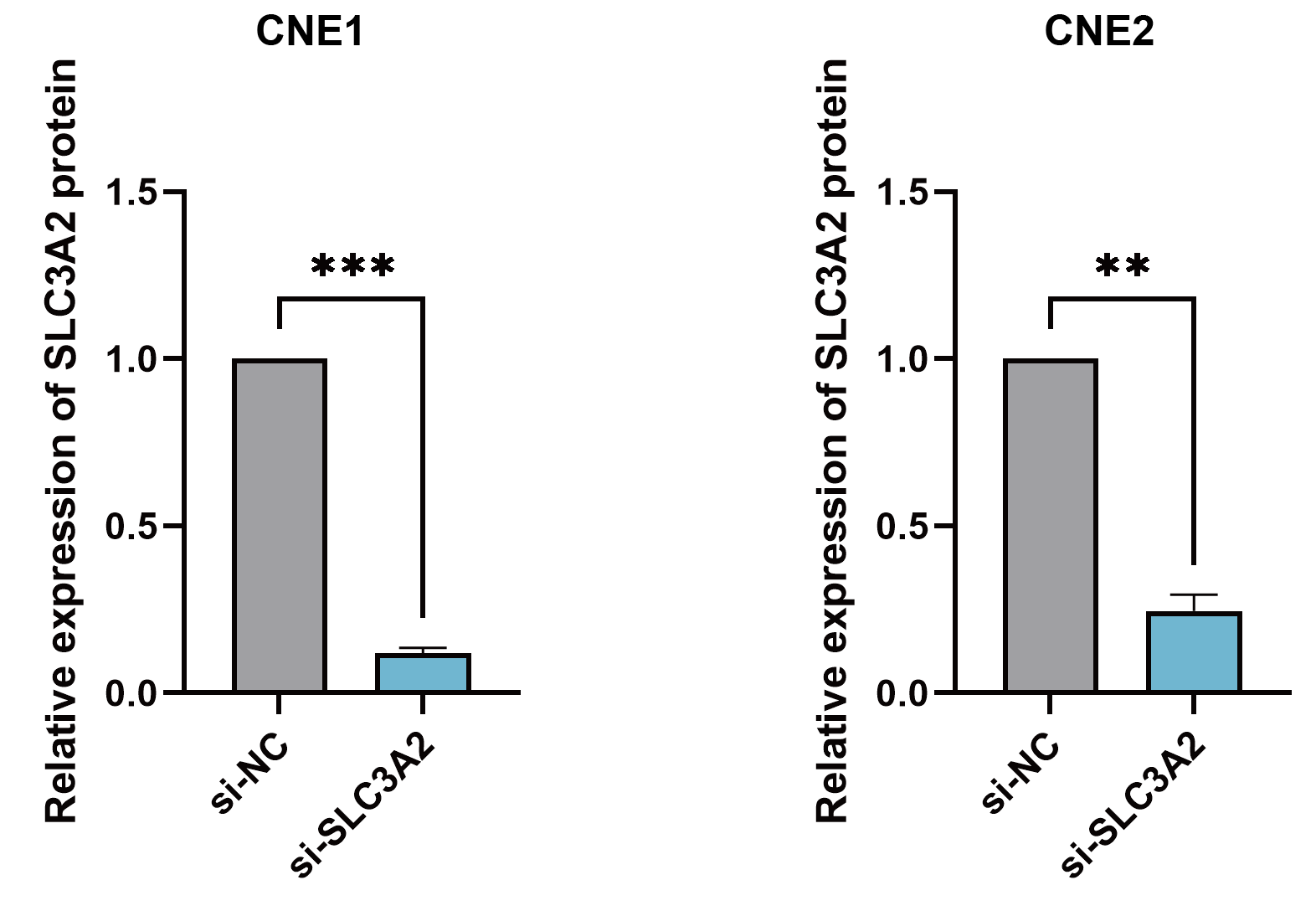


**Supplementary Figure 2**. The protein bands were analyzed using Image Lab (version 3.0) to quantify the gene knockdown efficiency at the translational level. The knock-down efficiency is greater than 70% after the strip gray detection. Related to **Figure 5A**.


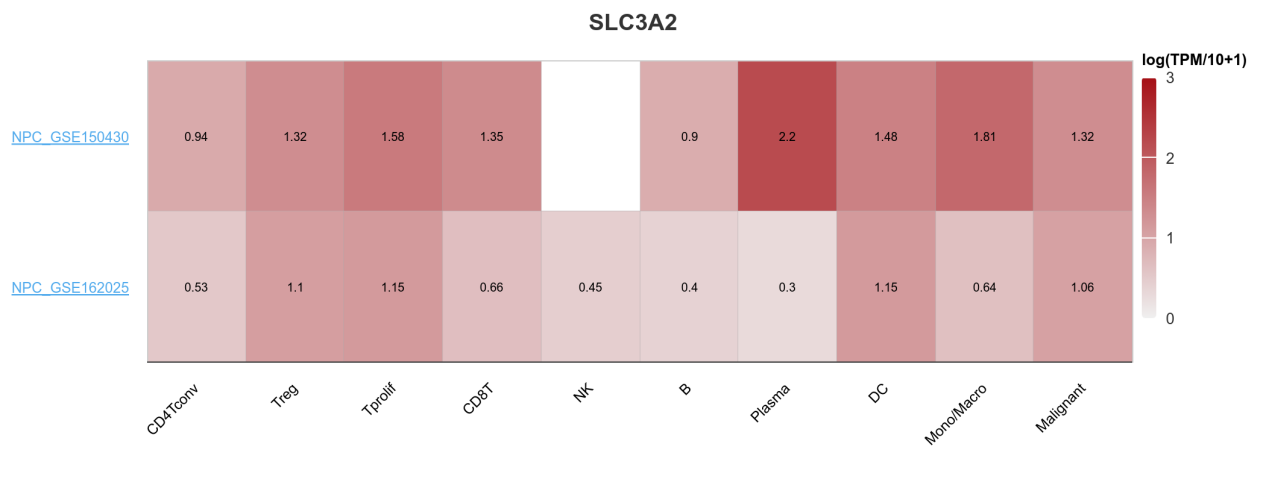


**Supplementary Figure 3**. The expression of SLC3A2 in TME-associated cells was investigated using two nasopharyngeal carcinoma datasets (GSE150430 and GSE162025) from the TISCH database. The results showed that SLC3A2 was expressed in both malignant tumour cells and immune cells.

**
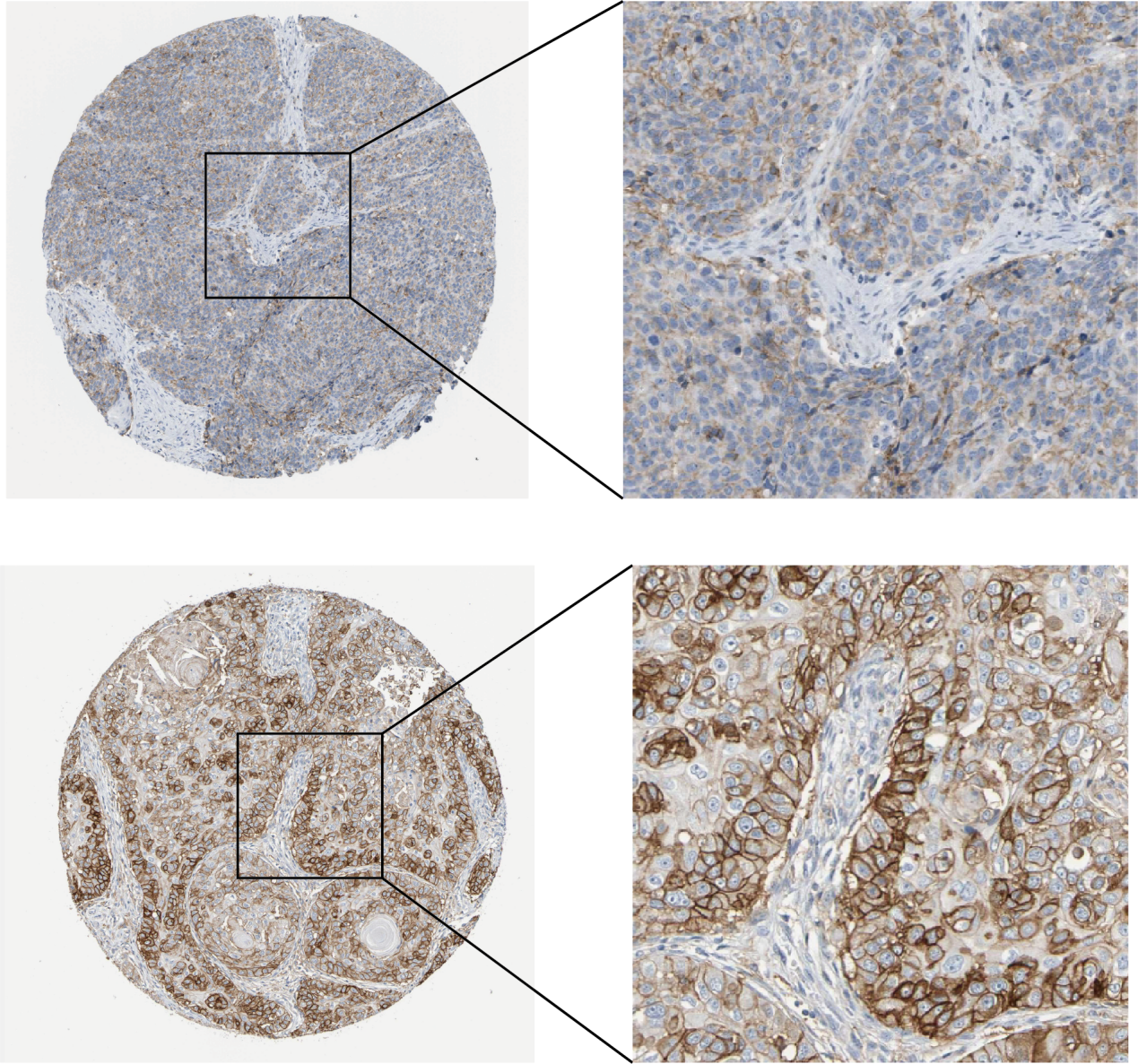
**

**Supplementary Figure 4**. Immunohistochemical data from the Human Protein Atlas database show that SLC3A2 is concentrated in tumour cell membranes.


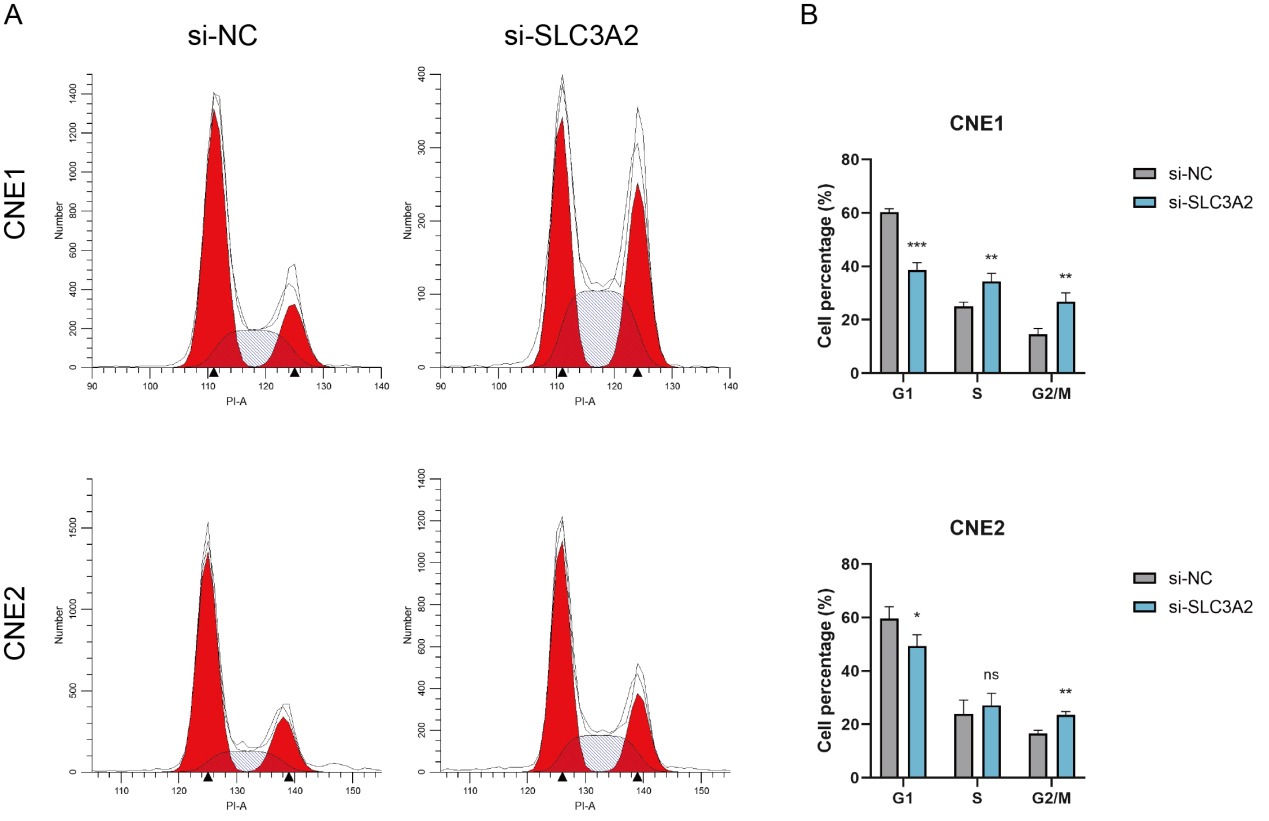


**Supplementary Figure 5**. SLC3A2 depletion leads to delayed S-phase. Cell cycle distribution measured by flow cytometry using propidium iodide (PI) staining. 10,000 cells/condition were analysed. A: A representative cell cycle profile of the control and SLC3A2 knockdown cells. B: The diagram of cell cycle distribution shows the percentage of G1, S, and G2/M phase cells in the two groups. n

=

3.
